# Deletion Involving the 7q31-32 Band at the CADPS2 Gene Locus in a Patient with Autism Spectrum Disorder and Recurrent Psychotic Syndrome Triggered by Stress

**DOI:** 10.1155/2017/4254152

**Published:** 2017-10-19

**Authors:** Paulo André Pera Grabowski, Alexandre Ferreira Bello, Diogo Lima Rodrigues, Murilo José Forbeci, Vinicius Motter, Salmo Raskin

**Affiliations:** ^1^Psychiatric Outpatient Unit, The Municipal Health Department of São José dos Pinhais, São José dos Pinhais, PR, Brazil; ^2^Pontifical Catholic University of Paraná, Faculdade Evangélica do Paraná, and Universidade Positivo, Curitiba, PR, Brazil; ^3^Evangelical Hospital, Hospital Nossa Senhora das Graças, Vita Batel Hospital, and Hospital Pequeno Príncipe of Curitiba, Curitiba, PR, Brazil

## Abstract

Autism spectrum disorder (ASD) is a neurodevelopmental disorder marked by impairments in social functioning, language, communication, and behavior. Recent genome-wide association studies show some microdeletions on the 7q31-32 region, including the CADPS2 locus in autistic patients. This paper reports the case of a patient with ASD and recurrent psychotic syndrome, in which a deletion on the 7q31-32 band at the CADPS2 gene locus was evidenced, as well as a brief review of the literature on the CADPS2 gene and its association with ASD.

## 1. Introduction

Autism is a neurodevelopmental disorder marked by impairments in social functioning, language, communication, and behavior. Epidemiological studies show that the prevalence of autistic spectrum disorders (ASDs) is 3–6/10,000, with a ratio of 3 men to each woman. The concordance rates in monozygotic and dizygotic twins are about 90% and 10%, respectively, suggesting a strong genetic component [1]. Cytogenetic studies have shown alterations in chromosomes 2, 3, 4, 5, 7, 8, 11, 13, 15, 16, 17, 19, 22, and *X*, including deletions, duplications, translocations, and inversions [2]. Several mutations have already been linked with autism and include, for example, the CYFIP1M, GABRB3, and UBE3A genes [3]. Mutations in the CADPS2 gene have also been associated with the disorder [4].

The activating protein family, which depends on calcium for secretion, consists of two members: CADPS and CADPS2 (Ca^2+^-dependent activator protein for secretion 2) [5–7]. Studies indicate that CADPS is involved in vesicle release during exocytosis [5, 8–10]. CADPS2 is immunohistochemically related to the release of brain-derived neurotrophic factor in various regions of the brain in mice. CADPS2 mRNA is found in several mice tissues, predominantly in the brain [11]. CADPS2 in humans is located on chromosome 7q31-32 at the AUTS1 locus [12]. Recent genome-wide association studies show some microdeletions on 7q31-32, including the locus of CAPS2, in autistic patients [12–14]. The presence of a de novo copy number variation due to microdeletions in the region containing CADPS2 has also been reported in some autistic patients [1]. The search for genomic alterations by microarray has already been suggested as the first line of genetic investigation in patients with autism [2, 3, 15].

One study analyzed the effects of CADPS2 knockout in mice exposed to an eight-arm radial maze in which these mice had decreased locomotor activity and fewer entries into the arms [4]. This is the same change which was observed in rats that received the D2-dopaminergic receptor agonist LY171555. Since the proteins of the CADPS family interact with the D2-dopaminergic receptor, the authors concluded that changes due to CADPS2 knockout could be associated with dopaminergic pathways [16]. A case of deletion involving the 7q31-32 band at the CADPS2 gene locus is presented in a patient with ASD.

## 2. Case Report

A male patient, aged 32 years, sought psychiatric care for psychotic symptoms. At that time he worked as a doorman and misinterpreted the intentions of a resident of the building, imagining that she was in love with him. He approached the resident in her apartment and she was startled, verbally assaulting him. The effects of this encounter began to manifest in total insomnia. In a few days he presented symptoms of acute confusion, with behavioral disorganization (throwing himself on the floor and standing around doors for no apparent reason), psychomotor agitation, time disorientation, disturbances in recent memory, irritability, hostility, loose association of ideas, and delusions (e.g., that the army was after him for not having enlisted at the proper age). These symptoms remitted after 3 months of clozapine therapy 200 mg/day (olanzapine and aripiprazole having had no effect). The patient is currently not on antipsychotic medication.

The patient's clinical history has been marked by poor social performance. He has often been mistaken about the intentions of girls at the psychosocial center he usually attends, which has become his only social activity. He reports occasional derogatory auditory hallucinations and hyperesthesia in the scalp. When he develops insomnia or more frequent hallucinations, he takes clozapine (200 mg/day).

He was born, without complications, by cesarean section. He first spoke at the age of three and has always been withdrawn socially. In childhood he had untreated attention deficit symptoms and failed three years of elementary school. At the age of 10, he began experiencing bilateral hand tremors and difficulties with fine motor coordination, for which he began physiotherapy and consulted a neurologist. His mother has always characterized him as clumsy. At age 18, he began having delusions, auditory hallucinations, increasing social isolation, and behavioral disorganization, for which he was hospitalized. At age 28, he was diagnosed with a psychotic depressive episode (with derogatory auditory hallucinations). His symptoms improved with use of risperidone, and for the next 4 years he remained asymptomatic without medication.

**Figure 1:**
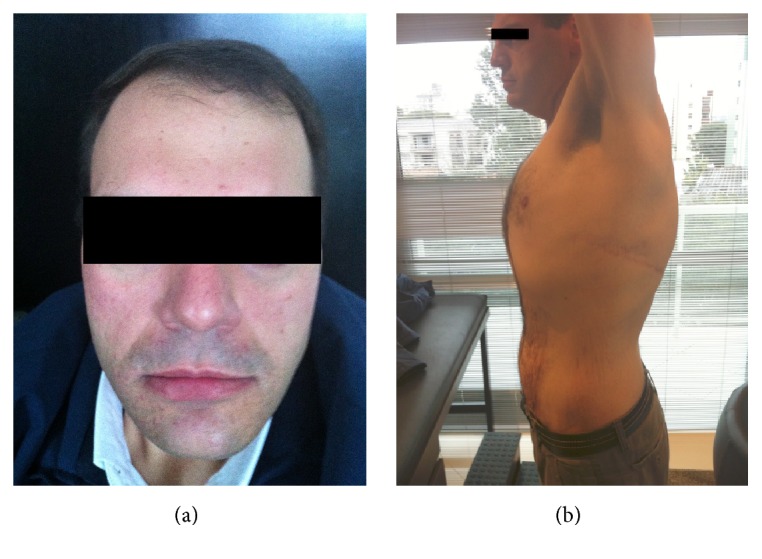
(a) Patient's face showing broad forehead, high hair implantation, and light hypertelorism; (b)* Pectus carinatum*.

**Figure 2:**
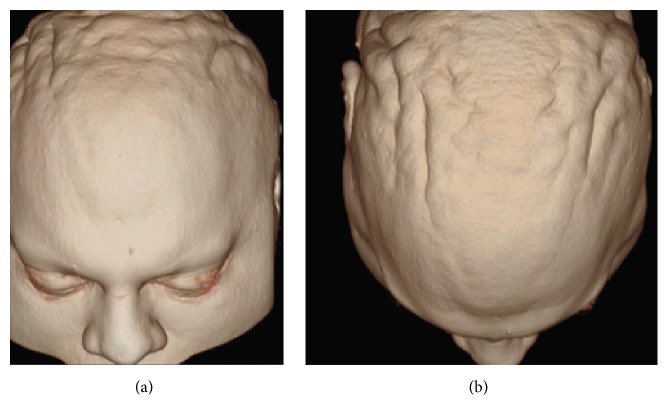
((a) and (b)) Presence of* cutis verticis gyrata* proven in this MRI image.

In physical examination at age 32, he presented bilateral positional and intention hand tremors, which were worse on the left side, hypertelorism, broad forehead and high hair implantation (Figure 1(a)),* pectus carinatum* (Figure 1(b)), scalp changes compatible with* cutis verticis gyrata* (Figure 2(a)), and a slight imbalance in tandem gait.

**Figure 3:**
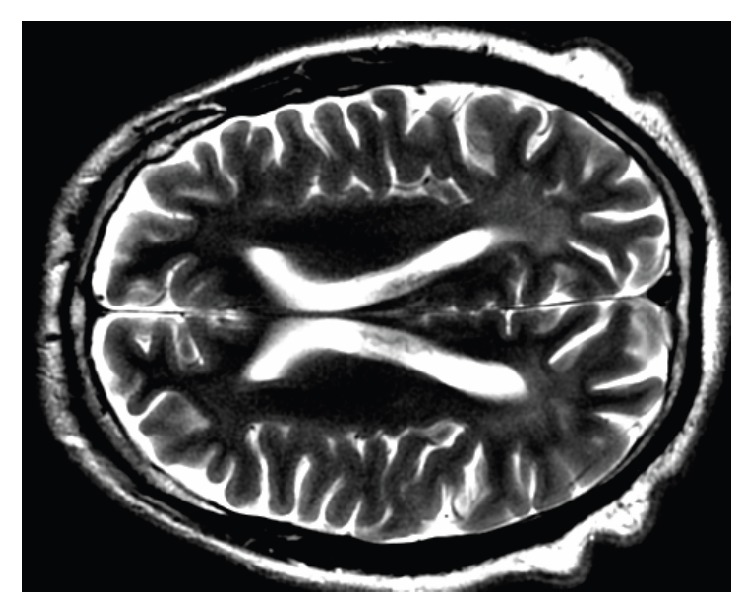
Bilateral hyperintensities in T2 in the occipitoparietal white matter.

We did not apply psychological tests for the diagnosis of autism; however, we used the diagnostic criteria of DSM-5 299.00 with level of severity one and intellectual disability. MRI revealed anteriorization and enlargement of the anterior-posterior diameter of the frontal sinus, reduction of the maxillary antrum (with hypopneumatization), scalp anomaly compatible with cutis verticis gyrata, probable small cyst of the pineal gland, subtle proptosis of the eyes, prominent cerebrospinal fluid spaces along the optic nerve sheaths, reductions in brain parenchyma incompatible with age (prominent in the posterior parietal regions), and FLAIR hyperintensities of white matter in the occipital horns, atria, posterior horns of the lateral ventricles, part of the corona radiata, and semioval centers, as well as in deep white matter of the superior, middle, and inferior frontal gyrus, the postcentral gyrus, and the upper and lower parietal lobes (Figure 3). These changes in white matter presented no restrictions on diffusion or increased signal intensity with contrast enhancement, which raised the possibility of metabolic changes after discussion with the radiologist. Following this line of reasoning, we ordered adult tests for metabolic errors, beginning with those that could account for this image pattern, the physical examination, and the psychiatric history.

Due to the lesions with demyelinating characteristics, recurrent stress-triggered psychotic syndrome,* pectus carinatum*, and hypopneumatization of the sinus, the possibility of alpha-mannosidosis and Krabbe's disease was suggested. These hypotheses were excluded by the enzymatic assays. Due to the physical characteristics, psychiatric history, and* cutis verticis gyrata*, the possibility of a syndromic genetic disease was suggested.

**Figure 4:**
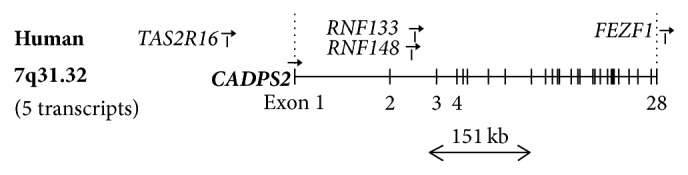
Exon-intron structure of the human CADPS2/CAPS2 gene located on 7q31.32 and the approximate location of the 151 kb mutation found in the case described.

The karyotype was normal. Comparative genomic hybridization by oligonucleotide arrangement (microarray) showed a deletion of approximately 151 kb in the long arm of chromosome 7, which involved band 7q31-32 (Figure 4). This deletion is intragenic in the CADPS2 gene, involving exons 3 through 9. Mutation searches performed in the patient's mother and sister were negative by the same method. Since the father is deceased, it could not be determined whether the patient had a de novo mutation.

## 3. Discussion

The investigation of this patient with lifelong autism and recurrent psychotic disorder secondary to stress proved the existence of an intragenic deletion of the CADPS2 gene. This mutation may be de novo or inherited from the father, since it was not possible to perform the examination in this relative.

The signs and symptoms of autism are consistent with previous descriptions of patients with this deletion. However, MRI findings and changes in the physical examination of this patient have not been described. Recurrent psychosis secondary to stress may be related to changes in dopaminergic receptors type D2, involved in the proteins activity of the CADPS2 family.

## 4. Conclusion

This report is based on a case of ASD associated with recurrent mental confusion episodes, which could easily be diagnosed as psychosis depending on where the individual is treated. An association between the protein secreted by the CADPS2 gene and the dopamine receptor may in some way be responsible for the patient's exaggerated sensitivity to the development of psychotic symptoms through stress (a known dopamine-triggering factor). There is an increasing need to publish cases featuring the clinical characteristics of patients diagnosed with psychiatric syndromes in which the possible contributions of specific mutations can be discussed, so that clinicians can narrow their diagnosis through specific phenotypic characteristics.

## References

[1] Sadakata T., Furuichi T. (2010). Ca^2+^-dependent activator protein for secretion 2 and autistic-like phenotypes. *Neuroscience Research*.

[2] Miles J. H. (2011). Autism spectrum disorders—a genetics review. *Genetics in Medicine*.

[3] Liu X., Takumi T. (2014). Genomic and genetic aspects of autism spectrum disorder. *Biochemical and Biophysical Research Communications*.

[4] Sadakata T., Washida M., Iwayama Y. (2007). Autistic-like phenotypes in Cadps2-knockout mice and aberrant CADPS2 splicing in autistic patients. *The Journal of Clinical Investigation*.

[5] Berwin B., Floor E., Martin T. F. J. (1998). CAPS (mammalian UNC-31) protein localizes to membranes involved in dense-core vesicle exocytosis. *Neuron*.

[6] Speidel D., Varoqueaux F., Enk C. (2003). A family of Ca^2+^-dependent activator proteins for secretion: Comparative analysis of structure, expression, localization, and function. *The Journal of Biological Chemistry*.

[7] Sadakata T., Mizoguchi A., Sato Y. (2004). The Secretory Granule-Associated Protein CAPS2 Regulates Neurotrophin Release and Cell Survival. *The Journal of Neuroscience*.

[8] Renden R., Berwin B., Davis W. (2001). Drosophila CAPS is an essential gene that regulates dense-core vesicle release and synaptic vesicle fusion. *Neuron*.

[9] Tandon A., Bannykh S., Kowalchyk J. A., Banerjee A., Martin T. F. J., Balch W. E. (1998). Differential regulation of exocytosis by calcium and CAPS in semi- intact synaptosomes. *Neuron*.

[10] Sadakata T., Shinoda Y., Sekine Y. (2010). Interaction of Calcium-dependent Activator Protein for Secretion 1 (CAPS1) with the class II ADP-ribosylation factor small GTPases is required for dense-core vesicle trafficking in the trans-Golgi network. *The Journal of Biological Chemistry*.

[11] Sadakata T., Itakura M., Kozaki S., Sekine Y., Takahashi M., Furuichi T. (2006). Differential distributions of the Ca^2+^-dependent activator protein for secretion family proteins (CAPS2 and CAPS1) in the mouse brain. *Journal of Comparative Neurology*.

[12] International Molecular Genetic Study of Autism Consortium (IMGSAC) (2001). Further characterization of the autism susceptibility locus AUTS1 on chromosome 7q. *Human Molecular Genetics*.

[13] Christian S. L., Brune C. W., Sudi J. (2008). Novel submicroscopic chromosomal abnormalities detected in autism spectrum disorder. *Biological Psychiatry*.

[14] Szatmari P. (2007). Mapping autism risk loci using genetic linkage and chromosomal rearrangements. *Nature Genetics*.

[15] Shen Y. (2010). Clinical genetic testing for patients with autism spectrum disorders. *Pediatrics*.

[16] Binda A. V., Kabbani N., Levenson R. (2005). Regulation of dense core vesicle release from PC12 cells by interaction between the D2 dopamine receptor and calcium-dependent activator protein for secretion (CAPS). *Biochemical Pharmacology*.

